# Supplementary material for: Iterative improvement in the automatic modular design of robot swarms
Source: PeerJ Comput Sci. 2020 Dec 7;6:e322. doi: 10.7717/peerj-cs.322 (PMC7924708; doi:10.7717/peerj-cs.322)
Supplement: Supplemental Information 3 [file peerj-cs-06-322-s003.zip › argos3/doc/api/standalone/a00412_source.html]

ARGoS: plugins/robots/e-puck/simulator/dynamics2d\_epuck\_model.cpp Source File


- Main Page
- Related Pages
- Namespaces
- Classes
- Files

- File List
- File Members

# plugins/robots/e-puck/simulator/dynamics2d\_epuck\_model.cpp

Go to the documentation of this file.

```
00001 
00007 #include "dynamics2d_epuck_model.h"
00008 #include <argos3/plugins/simulator/physics_engines/dynamics2d/dynamics2d_gripping.h>
00009 #include <argos3/plugins/simulator/physics_engines/dynamics2d/dynamics2d_engine.h>
00010 
00011 namespace argos {
00012 
00013    /****************************************/
00014    /****************************************/
00015 
00016    static const Real EPUCK_MASS                = 0.4f;
00017 
00018    static const Real EPUCK_RADIUS              = 0.035f;
00019    static const Real EPUCK_INTERWHEEL_DISTANCE = 0.053f;
00020    static const Real EPUCK_HEIGHT              = 0.086f;
00021 
00022    static const Real EPUCK_MAX_FORCE           = 1.5f;
00023    static const Real EPUCK_MAX_TORQUE          = 1.5f;
00024 
00025    enum EPUCK_WHEELS {
00026       EPUCK_LEFT_WHEEL = 0,
00027       EPUCK_RIGHT_WHEEL = 1
00028    };
00029 
00030    /****************************************/
00031    /****************************************/
00032 
00033    CDynamics2DEPuckModel::CDynamics2DEPuckModel(CDynamics2DEngine& c_engine,
00034                                                 CEPuckEntity& c_entity) :
00035       CDynamics2DSingleBodyObjectModel(c_engine, c_entity),
00036       m_cEPuckEntity(c_entity),
00037       m_cWheeledEntity(m_cEPuckEntity.GetWheeledEntity()),
00038       m_cDiffSteering(c_engine,
00039                       EPUCK_MAX_FORCE,
00040                       EPUCK_MAX_TORQUE,
00041                       EPUCK_INTERWHEEL_DISTANCE),
00042       m_fCurrentWheelVelocity(m_cWheeledEntity.GetWheelVelocities()) {
00043       /* Create the body with initial position and orientation */
00044       cpBody* ptBody =
00045          cpSpaceAddBody(GetDynamics2DEngine().GetPhysicsSpace(),
00046                         cpBodyNew(EPUCK_MASS,
00047                                   cpMomentForCircle(EPUCK_MASS,
00048                                                     0.0f,
00049                                                     EPUCK_RADIUS + EPUCK_RADIUS,
00050                                                     cpvzero)));
00051       const CVector3& cPosition = GetEmbodiedEntity().GetOriginAnchor().Position;
00052       ptBody->p = cpv(cPosition.GetX(), cPosition.GetY());
00053       CRadians cXAngle, cYAngle, cZAngle;
00054       GetEmbodiedEntity().GetOriginAnchor().Orientation.ToEulerAngles(cZAngle, cYAngle, cXAngle);
00055       cpBodySetAngle(ptBody, cZAngle.GetValue());
00056       /* Create the body shape */
00057       cpShape* ptShape =
00058          cpSpaceAddShape(GetDynamics2DEngine().GetPhysicsSpace(),
00059                          cpCircleShapeNew(ptBody,
00060                                           EPUCK_RADIUS,
00061                                           cpvzero));
00062       ptShape->e = 0.0; // No elasticity
00063       ptShape->u = 0.7; // Lots of friction
00064       /* Constrain the actual base body to follow the diff steering control */
00065       m_cDiffSteering.AttachTo(ptBody);
00066       /* Set the body so that the default methods work as expected */
00067       SetBody(ptBody, EPUCK_HEIGHT);
00068    }
00069 
00070    /****************************************/
00071    /****************************************/
00072 
00073    CDynamics2DEPuckModel::~CDynamics2DEPuckModel() {
00074       m_cDiffSteering.Detach();
00075    }
00076 
00077    /****************************************/
00078    /****************************************/
00079 
00080    void CDynamics2DEPuckModel::Reset() {
00081       CDynamics2DSingleBodyObjectModel::Reset();
00082       m_cDiffSteering.Reset();
00083    }
00084 
00085    /****************************************/
00086    /****************************************/
00087 
00088    void CDynamics2DEPuckModel::UpdateFromEntityStatus() {
00089       /* Do we want to move? */
00090       if((m_fCurrentWheelVelocity[EPUCK_LEFT_WHEEL] != 0.0f) ||
00091          (m_fCurrentWheelVelocity[EPUCK_RIGHT_WHEEL] != 0.0f)) {
00092          m_cDiffSteering.SetWheelVelocity(m_fCurrentWheelVelocity[EPUCK_LEFT_WHEEL],
00093                                           m_fCurrentWheelVelocity[EPUCK_RIGHT_WHEEL]);
00094       }
00095       else {
00096          /* No, we don't want to move - zero all speeds */
00097          m_cDiffSteering.Reset();
00098       }
00099    }
00100 
00101    /****************************************/
00102    /****************************************/
00103 
00104    REGISTER_STANDARD_DYNAMICS2D_OPERATIONS_ON_ENTITY(CEPuckEntity, CDynamics2DEPuckModel);
00105 
00106    /****************************************/
00107    /****************************************/
00108 
00109 }
```

---

Generated on 10 Jul 2018 for ARGoS by 
 1.6.1
